# On the characterization of the heterogeneous mechanical response of human brain tissue

**DOI:** 10.1007/s10237-016-0860-8

**Published:** 2016-12-08

**Authors:** Antonio E. Forte, Stephen M. Gentleman, Daniele Dini

**Affiliations:** 1 Department of Mechanical Engineering, Imperial College London, London, SW7 2AZ UK; 2 Department of Medicine, Imperial College London, London, W12 0NN UK

**Keywords:** Human brain tissue, Mechanical properties, Abaqus, FE modelling, Humidity and temperature, Heterogeneous

## Abstract

The mechanical characterization of brain tissue is a complex task that scientists have tried to accomplish for over 50 years. The results in the literature often differ by orders of magnitude because of the lack of a standard testing protocol. Different testing conditions (including humidity, temperature, strain rate), the methodology adopted, and the variety of the species analysed are all potential sources of discrepancies in the measurements. In this work, we present a rigorous experimental investigation on the mechanical properties of human brain, covering both grey and white matter. The influence of testing conditions is also shown and thoroughly discussed. The material characterization performed is finally adopted to provide inputs to a mathematical formulation suitable for numerical simulations of brain deformation during surgical procedures.

## Introduction

Synthetic phantoms and virtual models of human brain have achieved considerable results in several fields, from the mimicking of the electrophysiological functions of the cortical tissue in vitro (Tang-Schomer et al. 2014) to the simulation of the mammalian thalamocortical system (Izhikevich and Edelman 2008) and the reproduction of highly resolved 3D human brain models (Amunts et al. 2013). Equally important, and of particular interest for the study presented here, is the design of realistic mechanical models to capture human brain deformations; such models are not only to reproduce accurate geometries and structures but also to provide material formulations that could accurately reproduce the mechanical behaviour of such a complex organ. In this regard, a meticulous mechanical characterization provides valuable data for brain tissue modelling needed to perform, i.e. traumatic brain injury simulations (Brands et al. 2004; Nicolle et al. 2004) and biomechanics simulations and robotic surgery (Dumpuri et al. 2007; Miller 1999; Miller and Chinzei 1997, 2002; Miller et al. 2000). Furthermore, mechanical data on human brain tissue are important for the design of synthetic materials that can mimic the mechanical behaviour of the native tissues (Forte et al. 2016; Leibinger et al. 2015; O’Brien 2011) and promote cell differentiation and regeneration (Discher et al. 2009; Engler et al. 2008; Saha et al. 2008).

The mechanical characterization of brain tissue has been the object of many studies for over 50 years. However, due to the extreme complexity of the soft tissue and the wide range of experimental methods and protocols that have been used for its characterization, the results described in the literature often differ by orders of magnitude (Chatelin et al. 2010).

Animal brain tissue is widely used as a substitute for human brain tissue because of the high degree of similarity in terms of anatomical structures and mechanical properties (Nicolle et al. 2004). However, studies have shown substantial differences between human and animal brain tissue. In particular, Takhounts et al. (2003) and Prange and Margulies (2002) showed that human tissue is 40% stiffer than bovine and 29% stiffer than porcine tissues, respectively, by means of shear relaxation tests. Galford and McElhaney (1970) demonstrated that storage, loss and shear moduli are, respectively, 1.4, 2 and 1.5 times higher for monkey brain tissue than for humans. In contrast, Nicolle et al. (2004) concluded that human tissue has a higher storage modulus but similar loss modulus if compared with porcine brain tissue. Given these discrepancies and uncertainties in mapping data from animal to human tissues, there is a strong need for human brain issue testing. However, (1) only a few studies provide human brain tissue data and (2) the spread in these data undermines the achievement of an accurate mechanical characterization of the human brain tissue. For example, it is still not clear whether differences in post-mortem times cause variation in the mechanical response of the tissue; while some researchers show no (Budday et al. 2015; Darvish and Crandall 2001; McElhaney et al. 1973) or limited (Nicolle et al. 2005; Shen et al. 2006) changes in the measured properties due to different post-mortem times, Metz et al. (1970) reported changes from live to 45 min post-mortem times and Garo et al. (2007) showed an increase in the tissue stiffness in the first 6 h post-mortem.

Furthermore, the heterogeneity of the tissue is often neglected when analysing its mechanical properties (Bilston 2003; Bilston et al. 1997, 2001; Miller 1999; Miller and Chinzei 1997, 2002; Miller et al. 2000), mostly because of the difficulties in cutting and testing small samples. Nevertheless, Prange et al. found grey matter to be about 1.3 times stiffer than white matter (Prange and Margulies 2002; Prange et al. 2000), a finding confirmed by Nicolle et al. (2004). However, it should be noticed that in these last studies the grey matter was harvested from thalamus and that the mechanical properties of the brain tissue are likely to be affected by interregional heterogeneities (Coats and Margulies 2006; Elkin et al. 2011). In fact, grey matter coming from cortex seems to be less stiff than white matter according to other studies (Budday et al. 2015; Feng et al. 2013; Kaster et al. 2011; van Dommelen et al. 2010). These results are also supported by recent works on magnetic resonance elastography (MRE) that observed white matter shear modulus to be 1.2–2.6 times higher than that of grey matter (Bayly et al. 2012; Chatelin et al. 2010; Clayton et al. 2012). A few studies have also investigated the transversally anisotropic behaviour of the white matter in porcine and ovine brain tissue (Feng et al. 2013, 2017).

Differences in testing conditions and protocols are also responsible for the large variations in the results reported by different research groups. Gefen and Margulies (2004) argued an underestimation of the initial and long-term shear modulus due to a preconditioning of the tissue when tested in vitro. Similar results had been shown by Miller et al. (2000). Prevost et al. (2011b) noticed the indentation response on porcine brain tissue to be significantly stiffer in situ than in vivo, which is only in partial agreement with the previous findings by Gefen and Margulies (2004). Furthermore, they showed the in vivo and the in vitro response of the tissue to be similar, disagreeing with previous studies. As the authors underline, the braincase is a complex geometry and might play a significant role in the way it constrains the “incompressible” tissue during in vivo testing. Moreover, Chatelin et al. (2010) observed that the variation of results is also noticeable for a non-invasive in vivo technique such as MRE. Therefore, differences in testing protocols and conditions (when conducting in vitro testing) and/or equipment/transducers (in MRE) might be the principal cause of such a huge disparity in the results throughout the literature, also among similar species. For these reasons, understanding the role of these factors could lead to better measurements and consequentially to more accurate biomechanical models.

Compression tests have been widely used to characterize the mechanical behaviour of brain tissue and its rate dependency (Cheng and Bilston 2007; Franceschini et al. 2006; Miller and Chinzei 1997; Prevost et al. 2011a; Rashid et al. 2012; Shen et al. 2006). The majority of the studies used cylindrical samples and lubricant at the sample–plates interface to eliminate friction effects. Humidity conditions are usually not taken into consideration because of the short test duration (at least at high strain rates) and most of the tests are run at room temperature. Varying the temperature in a range of a few degrees (22–37 $^{\circ }\hbox {C}$, respectively, room and body temperature) does not seem to play a major role in the tissue response under compression if the tissue has been preserved at ice-cold temperature (4 $^{\circ }\hbox {C}$) before testing (Rashid et al. 2012). The use of different preservation temperatures might instead affect the results significantly (Rashid et al. 2012). In addition, difficulties in handling the tissue limit the ability to obtaining small size samples; this causes uncertainties due to possible heterogeneities of the tissue samples (white and grey matter present in unknown ratio) and lack of repeatability in terms of sample shapes. For this reason, some authors (Miller and Chinzei 1997, 2002; Rashid et al. 2012, 2013) decided to focus on the behaviour of the tissue as a bulk material. These studies used large samples (e.g. 30 mm diameter, 13 mm height) that included white and grey matter and blood vessels and, in certain cases, also the arachnoid membrane and the structure of the sulci (Miller and Chinzei 1997). However, isolating the brain matter from the meninges and testing grey and white matter separately could lead to a more precise material characterization and accurate modelling.

Dynamic mechanical analysis (DMA) testing in shear is considered a standard method to measure the linear viscoelastic behaviour of the tissue. This is limited to a 1% applied strain for the brain tissue (Brands et al. 1999; Nicolle et al. 2004; Peters et al. 1997; Shen et al. 2006). Nevertheless, scientists have also tested brain tissue subjected to large strains in shear and high strain rates with custom-built mechanical testing devices; these studies were aimed at characterizing the response of the tissue undergoing impact loading (Donnelly and Medige 1997).

Although the effect of temperature is important in this kind of test, to the best of our knowledge, only a few studies have investigated this aspect (Brands et al. 2000; Hrapko et al. 2008; Peters et al. 1997; Shen et al. 2006), showing a decrease in the tissue stiffness due to increased temperatures.

Another important aspect to be considered when testing organic tissue is the prevention of the dehydration of the sample. Most of the studies have used moist chambers for testing brain tissue in DMA (Arbogast and Margulies 1997; Garo et al. 2007; Hrapko et al. 2006, 2008; Nicolle et al. 2004, 2005; Peters et al. 1997; Thibault and Margulies 1998; Vappou et al. 2007). Others have made use of silicone oil/adhesive (Fallenstein et al. 1969; Shen et al. 2006) or petroleum jelly (Bilston 2003; Bilston et al. 1997, 2001) applied to the exposed rim of the sample. There are also a few works in which humidity monitoring has not been mentioned/adopted (Brands et al. 1999; Darvish and Crandall 2001; Shuck and Advani 1972). Understanding the variability of the results in relation to humidity might also lead to a better interpretation of the available data in the literature as shown in one study on kidney tissue (Nicolle and Palierne 2010).

Regarding the specimen–plates interface, the majority of the studies have reported the use of sand paper attached to the plates of the equipment in order to avoid slipping (Arbogast and Margulies 1997; Bilston et al. 1997, 2001; Brands et al. 1999, 2000, 2004; Garo et al. 2007; Hrapko et al. 2006, 2008; Prange and Margulies 2002; Shen et al. 2006; Thibault and Margulies 1998) while others glued the specimen directly on the plates (Darvish and Crandall 2001; Nicolle et al. 2004, 2005; Takhounts et al. 2003). No difference in the results was observed using either methods (Brands et al. 2000). Nicolle et al. (2005) showed that when the samples were not glued to the plates and no sand paper was used to assure grip, the dynamic modulus was significantly affected by the sample height.

A summary of methods and conditions used in previous studies is given in Table 1.

**Table 1:** Literature summary

References	Origin	Test (state)	TA	HC	HE
Arbogast and Margulies (1997)	P	D (vt)		M	
Bilston (2003) and Bilston et al. (1997, 2001)	B	D, SR (vt)		P	
Brands et al. (1999, 2000, 2004)	P	D, SR (vt)	$\checkmark$		
Cheng and Bilston (2007)	B	CR (vt)			
Darvish and Crandall (2001)	B	D (vt)			
Fallenstein et al. (1969)	M, H	D, I (vt, vv)		SO	
Feng et al. (2013, 2017)	P, O	I, S (vt)		SS	$\checkmark$
Franceschini et al. (2006)	H	C, T, Cr (vt)			
Galford and McElhaney (1970)	M, H	D, CR, Cr (vt)			
Garo et al. (2007)	P	D, S (vt)		M	
Gefen and Margulies (2004)	P	I (vt, vv, st)			
Hrapko et al. (2006, 2008)	P	D, SR (vt)	$\checkmark$	M	
McElhaney et al. (1973)	M, H	D, C, I (vt, vv)			
Metz et al. (1970)	M	E (vt, vv)			
Miller (1999), Miller and Chinzei (1997, 2002) and Miller et al. (2000)	P	C, T, I (vt, vv)			
Nicolle et al. (2004, 2005)	P, H	D, SR (vt)		M	$\checkmark$
Peters et al. (1997)	B	D, SR (vt)	$\checkmark$	M	
Prange et al. (2000) and Prange and Margulies (2002)	P, H	SR, CR (vt)			$\checkmark$
Prevost et al. (2011a, b)	P	D, CR, I (vt, vv, st)			
Rashid et al. (2012, 2013)	P	C, S (vt)	$\checkmark$		
Shen et al. (2006)	P	D, SR, C (vt)	$\checkmark$	SO	
Shuck and Advani (1972)	H	D (vt)			
Takhounts et al. (2003)	B, H	SR (vt)			
Thibault and Margulies (1998)	P	D (vt)		M	
Vappou et al. (2007)	P	D, I (vt)		M	

Sample origin: *P* porcine, *B* bovine, *O* ovine, *M* monkey, *H* human. State: *vt* in vitro, *vv* in vivo, *st* in situ. Test: *D* dynamic mechanical analysis, *SR* shear–relaxation, *CR* compression–relaxation, *I* indentation, *S* shear, *C* compression, *T* tension, *Cr* creep, *E* elastic expansion. *TA* temperature analysis. *HC* humidity control: *M* moist chamber, *SO* silicone oil/adhesive, *SS* moisturized with phosphate-buffered saline solution, *P* petroleum jelly. *HE* heterogeneous analysis

In conclusion, the mechanical characterization of brain tissue is a very complex task, not yet fully addressed. Different testing protocols and monitoring of environmental and testing conditions may have led to the large disparity in results reported by recent studies; hence, shedding light on the causes of this variation could provide key information for the development of a unified experimental protocol. Furthermore, whereas the majority of the studies focused on animals (porcine, calf, bovine), human results are quite rare and insufficient to theorize a reliable material formulation that can accurately replicate the mechanical behaviour of brain tissue. For this reason, mechanical testing on human brain is of primary importance.

The scope of the work presented in this manuscript is twofold: On the one hand, in this study we address the effects of different testing conditions (such as temperature, humidity and sample thickness) on the in vitro mechanical response of human brain by means of oscillatory sweep frequency tests. Results obtained by compression–relaxation test are also presented and coupled with the rheological measurements in order to quantify the differences in stiffness between white and grey matter. This enables (1) the collection of human data by means of a heterogeneous mechanical characterization and (2) a methodological contribution towards understanding the importance of testing conditions when testing biological soft tissues. Furthermore, a comprehensive summary of the human brain tissue mechanical parameters and a mathematical material formulation that can be used to perform in silico experiments and predict the behaviour of the tissue under simulated surgical procedures is derived.

**Fig. 1:**
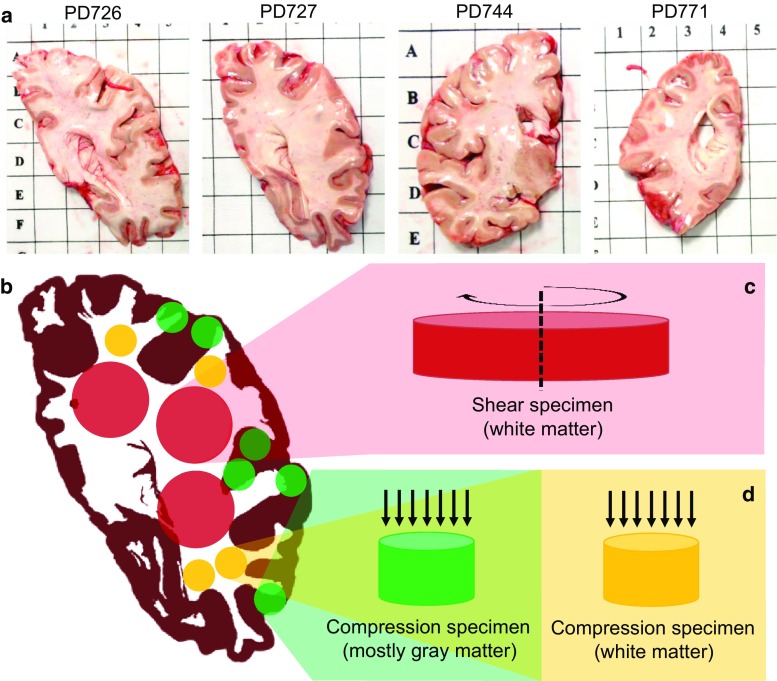
**a** P3 hemi-slices, different donors, **b** examples of locations for white matter- and grey matter-harvested specimens, **c** white matter specimen used for shear tests, **d** grey and white matter specimen used for compression–relaxation tests

## Material and methods

Tissue samples and associated clinical and neuropathological data were supplied by the Parkinson’s UK Brain Bank, funded by Parkinson’s UK, a charity registered in England and Wales (258197) and in Scotland (SC037554). We note that all procedures used by the tissue bank in the procurement, storage and distribution of tissue have been approved by the relevant Multicentre Research Ethics Committee (07/MRE09/72). Nine cases were supplied (examples of the hemi-slices in Fig. 1a). All the slices were cut from the parietal lobe because of its little use for Parkinson’s disease research. Lipp et al. (2013) observed that the reduction of brain elasticity in PD subjects reaches significance only in the lentiform nucleus. The parietal lobe is therefore considered as an area from which samples can be easily extracted for investigating the mechanical properties of human brain tissues with relevance that goes beyond PD subjects. A summary of the donors, including age, sex and post-mortem sample extraction, is reported in Table 2. Slices of the parietal lobe of the brain were collected from the brain tissue bank immediately after the release of the tissue (reported as post-mortem time in Table 2). The specimens were not frozen or kept in formalin at any time during this stage. They were stored in saline solution (PBS), kept at ice-cold temperature ($\sim$4 $^{\circ }\hbox {C}$) during transportation and tested within 2 h. White matter (25 ± 1 mm diameter, 7 ± 1 mm height) and grey matter (12 ± 1 mm diameter, 7 ± 1 mm height) cylindrical samples were punched out the hemi-slices using surgical trephines (Fig. 1c, d). Particular attention was paid in order to obtain homogeneous samples. The presence of circumvolutions and the limited thickness of the cortex reduce the availability of continuum tissue for cortical tissue samples. This is especially true in the proximity of a sulcus. However, the circumvolutions contribute to gather more grey matter in the gyri areas, which represent suitable spots for sample cutting. As a compromise, a small amount of white matter was often present in the grey matter samples.

**Table 2:** Summary of the donors

Case	Age	Gender	PM time (h)
PDC084	83	Male	26
PD726	65	Male	39
PD727	88	Male	26
PD728	80	Male	42
PD731	87	Male	48
PD744	81	Male	44
PD762	88	Male	36
PD771	81	Male	28
PD775	82	Female	48

From left to right: donor’s ID, age of the donor, gender, post-mortem time

DMA in shear is the most used method to measure the mechanical properties of the brain tissue. Moreover, rheometers are often equipped with (1) a secondary force sensor that allows the monitoring of the preloading conditions and (2) a temperature conditioning system on both lower and upper platens. The way the instrument is designed facilitates the use of moist chambers to control humidity. For all these advantages, the rheometer was chosen as primary instrument for investigating the variability in the measurements of white matter mechanical properties due to changes in the testing conditions. Unfortunately, difficulties in obtaining grey matter specimens with a reasonable shape resulted in a limit to the applicability of this methodology to the cerebral cortex. As previously mentioned, this is mainly due to the limited and peripheral amount of grey matter in the slices (Fig. 1b), which required a smaller trephine to extract homogeneous samples. As a consequence, the grey matter samples (see Fig. 1d) had an excessive height-to-diameter ratio (about 0.6), not suitable for rheometric tests. Therefore, in order to obtain a heterogeneous characterization of the tissue, the compression–relaxation test was chosen as testing protocol for the grey matter. Despite the lack of temperature control system on our equipment, this test can provide additional information on the response of the tissue to both small and large strains together with more insights into its rate dependency. A set of white matter samples was also tested in compression–relaxation in order to fully validate the results obtained from the rheological testing and compare the mechanical properties of white and grey matter over the same testing protocol.

### Rheometric tests on white matter

A Discovery HR-1 Rheometer (TA Instruments, Germany) was used to measure the storage $(G^{\prime })$ and the loss ($G^{{\prime }{\prime }})$ moduli of the white matter and to investigate the influence of testing conditions on the results. A plate–plate flat configuration was used (25 mm diameter), and sand paper was attached on the surface of the plates to prevent slipping. Each sample was gently wiped on the top and bottom surfaces before positioning, to avoid the formation of a thin film of PBS between the specimen and the sand paper. The top plate of the rheometer was slowly lowered until touching the top of the sample and measuring a maximum axial force of 0.01 N. A shear strain of 1% was applied on the upper plate varying the frequency from 0.01 to 25 Hz (sweep frequency analysis). For higher frequencies, the measurements were unreliable due to inertia effects. A waiting time of 120 s was set at the beginning of the test allowing the specimen to conform to the temperature imposed at the rheometer plates.

To avoid dehydration, a moist chamber was applied to the upper frame of the rheometer. The moist chamber consisted in disposable cylindrical superabsorbent paper towels kept wet by continuously spraying water using a syringe pump. Therefore, we assume that the condition inside the chamber produces a relative humidity close to 100%. Special care was taken assuring that the towel would not obstruct the moving parts of the testing rig. A number of 10 samples were tested using these conditions. The same test set-up was run without moist chamber for comparison (10 samples). In addition, a small set of samples (6 samples) was also tested applying petroleum jelly on the exposed rim of the brain specimen instead of using the moist chamber. The investigation was performed at controlled temperatures of 24 and 37 $^{\circ }\hbox {C}$. Four additional samples were tested using a temperature sweep analysis between 22 and 37 $^{\circ }\hbox {C}$ at 1.59 Hz (10 rad/s), with and without moist control. The effect of sample thickness was evaluated with a small set of dedicated samples (3 samples) of different heights (2, 5 and 8 mm) tested at 37 $^{\circ }\hbox {C}$ using the moist chamber.

### Compression–relaxation tests on grey matter

The Mach-1™ mechanical testing system (Biomomentum, Canada) was chosen as testing rig for the compression tests. A 1.5 N single-axis load cell was used to measure the vertical force (75 $\upmu$N resolution), and the vertical displacement was measured by the moving stage of the rig ($0.1\,\upmu$m resolution). Silicone oil with a kinematic viscosity of $5 \times 10^{-4}\,\hbox {m}^{2}/\hbox {s}$ was applied at the interface between the sample and the compression platens in order to minimize friction effects (Forte et al. 2015, 2016). No preconditioning was performed, and only one loading cycle was executed on each sample. The specimen was left for 1 min before the actual test began to obtain stable measurements of load and height and then compressed at constant velocity until a displacement corresponding to 30% of the measured height (corresponding to 0.356 true strain) was achieved. Afterwards, a relaxation step of 500 s was applied by holding the upper plate at the maximum strain value. The tests were conducted at three different velocities: “Fast”: 500 mm/min, “Medium”: 5 mm/min and “Slow”: 0.05 mm/min corresponding to the strain rates of about 1, $1 \times 10^{-2}$ and $1 \times 10^{-4}\,\hbox {s}^{-1}$, respectively. The data were acquired at a sample rate of 100 Hz. The moist chamber described in the previous section was exceptionally used for the lowest strain rate because of the considerable duration of the test (about 45 min). A total of 30 white matter samples and 30 grey matter samples were tested. For each matter type, samples were divided into three velocity groups, obtaining 10 specimens for each velocity.

**Fig. 2:**
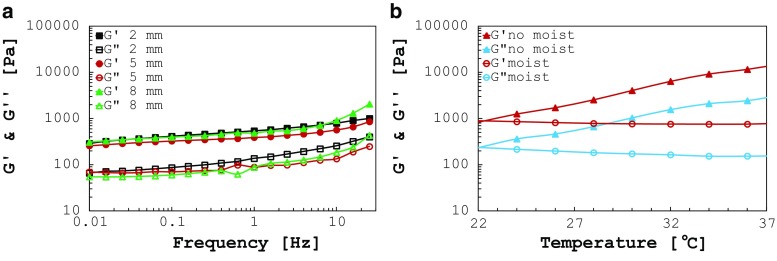
**a** Influence of sample thickness on storage and loss moduli. Tests were run at 37 $^{\circ }\hbox {C}$ using the moist chamber, and sand paper was used at the specimen–plate interfaces to avoid slipping. **b** Storage and loss moduli for two sweep temperature tests run consecutively on the same white matter sample with and without moist chamber at 1.59 Hz (10 rad/s)

**Fig. 3:**
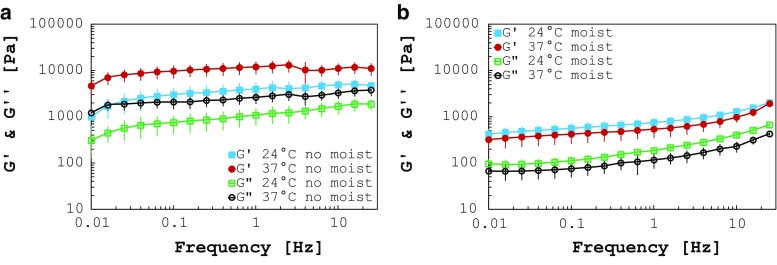
**a** Storage and loss moduli of human white matter tested at 24 and 37 $^{\circ }\hbox {C}$ with no humidity control. Human tissue shows higher moduli at the higher temperature, due to the faster rate of dehydration, **b** storage and loss moduli of human white matter tested at 24 and 37 $^{\circ }\hbox {C}$ with humidity control by using a moist chamber. The results show softening of the tissue at increasing temperatures

## Results and discussion

### Rheometric tests on white matter

#### Effect of sample thickness

Three white matter specimens with different heights (2, 5 and 8 mm, respectively) were tested by sweep frequency protocol at 37 $^{\circ }\hbox {C}$ using the moist chamber. Figure 2a shows that the results are not affected by the sample thickness when sand paper is used to assure grip between the specimen and the rheometer plates. The small spread in the curves might be caused by the slightly different shape of the sample and/or different orientation of white matter fibres in the specimens.

#### Effect of humidity and temperature

The increase in temperature leads to a faster dehydration of the sample if humidity is not kept at high levels. Figure 3a shows the error introduced in the measurements when humidity is not preserved, with respect to the same test run using the moist chamber (Fig. 3b, average and standard deviation bars) for 24 and 37 $^{\circ }\hbox {C}$. An increase of the storage and loss moduli is clearly visible for both the temperatures analysed. The stiffness of the tissue tends to rise proportionally with temperature when the moist chamber is not in place (see sweep temperature analysis below and results in Fig. 2b). In particular, choosing as example the frequency of 1.59 Hz, $G^{\prime }$ is about 5.2 times higher at 24 $^{\circ }\hbox {C}$ (4322 vs. 805 Pa, average values) and 21.9 times higher at 37 $^{\circ }\hbox {C}$ (12,544 vs. 572 Pa, average values) when humidity is not monitored. The same trends are noticeable for $G''.$ This is certainly due to dehydration of the tissue, enhanced at higher temperatures, that occurs immediately after the starting of the test, as shown by the first data points in Fig. 3a, b. The phenomenon was further investigated on four control samples by means of the sweep temperature analysis. Figure 2b depicts a sample plot of the results. Each specimen was first tested without moist chamber to observe the changes in the moduli with temperature. The increasing trend is clearly visible for $G'$ and $G''$ from 22 to 37 $^{\circ }$C. Afterwards, the specimen was rehydrated in PBS for 30 min and the same analysis was run using the moist chamber. The second time the curves assumed the correct trend, with the moduli decreasing over increasing temperature. The almost identical starting points of the curves in Fig. 2b exclude ageing effects in the test. In fact, if the specimens had aged during the first run they would not have been able to recover their original mechanical properties when rehydrated. Hence, the authors conclude that in case humidity is not controlled, the tissue undergoes a sudden dehydration and consequent stiffening, which is directly proportional to temperature. The results obtained under full humidity control are shown in Fig. 3b (average and standard deviation bars) where $G^{\prime }$ and $G^{{\prime }{\prime }}$ are, respectively, 1.4 and 1.6 times lower if measured at body temperature (37 $^{\circ }\hbox {C}$) rather than at room temperature (24 $^{\circ }\hbox {C}$).

**Fig. 4:**
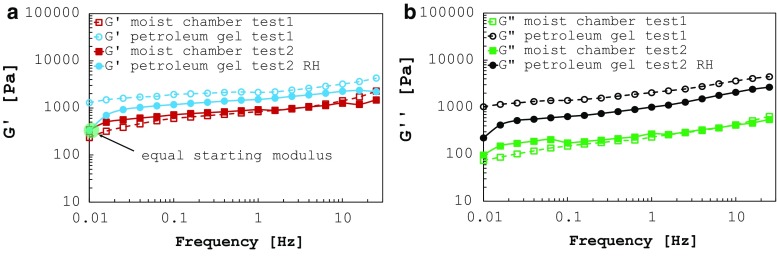
**a** Storage modulus and **b** loss modulus measured for two consecutive runs (using the moist chamber for the first and the petroleum jelly for the second run) on each sample. The second sample underwent rehydration between the two runs. Only two tests are reported for clarity

**Fig. 5:**
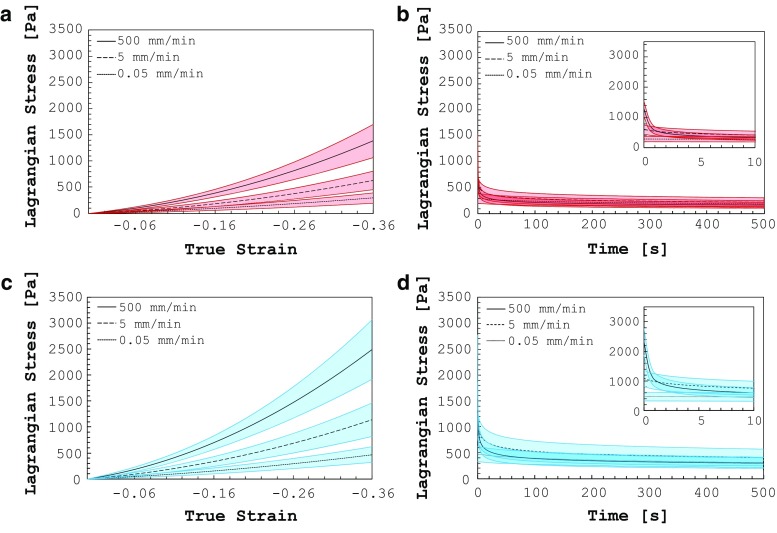
Compression and relaxation tests on human grey (**a**, **b**) and white matter (**c**, **d**) at three different displacement rates for an applied nominal strain of 0.3

Six additional samples were tested using alternately the moist chamber and petroleum jelly as dehydration preventers. For clarity, sample plots of the results are shown in Fig. 4. The specimens underwent the sweep frequency analysis at 37 $^{\circ }\hbox {C}$ for two times consecutively. For the second tests, preconditioning effects can be neglected since the applied strain is small (1%). Samples 1 and 2 are reported in Fig. 4 as examples. Firstly, sample 1 (namely test 1 in Fig. 4) was tested using the moist chamber. Afterwards, the chamber was removed and petroleum jelly applied to the exposed rim of the sample before running the test for the second time. As shown in Fig. 4, $G'$ rises about 2.5 times and $G''$ about 8.6 times when using petroleum jelly. Test 2 was run using the same procedure but rehydrating the specimen in PBS solution for 30 min after the first run. The results (Fig. 4, test 2 and test 2 RH) exhibit a closer match with the tests conducted using the moist chamber, but dehydration effects are still noticeable, with an increase of $G'$ and $G''$ at 1.59 Hz by 1.8 and 4.1 times, respectively. The measured value for $G'$ at the beginning of the test is identical for the two runs. However, a sudden increase in the storage modulus is clearly visible (Fig. 4a, test 2 RH). Tests 3, 4, 5 and 6 (not reported in Fig. 4) show the same trends. This proves that rehydrating the sample after the first run had the benefit of keeping the humidity at high levels within the tissue while the petroleum jelly was being applied on the exposed rim. On the other hand, even if it is certain that the jelly reduces the moisture loss during the test, its contribution is not sufficient in order to completely avoid dehydration. Moreover, an increase in $G''$ is still visible (also for the first data point, test 2 RH in Fig. 4b), probably due to a small damping introduced by the gel on the rheometer plates in addition to dehydration effects.

### Compression–relaxation tests on grey matter

Compression–relaxation tests on brain tissue confirmed its strong rate-dependent nature. Stresses in grey and white matters become about 4.7 and 5.3 times higher, respectively, when increasing the applied displacement rate from 0.05 to 500 mm/min. The averaged compression curves and the relative standard deviations are reported in Fig. 5a, c. The Lagrangian stress (or nominal stress) was calculated dividing the measured force by the initial area of the specimen (measured for each test). The true strain was calculated by recording the displacement over time during the compression step as shown in Forte et al. (2015). The relaxation curves along with the relative standard deviations (shaded areas) are shown in Fig. 5b, d. The material relaxation is clearly visible for both the white and grey matter with the stress value dropping immediately after the compression plate stops. White matter compressive stresses seem to be on average about 1.8 times higher than grey matter when comparing them over the three different strain rates.

### Comparison between testing protocols

**Fig. 6:**
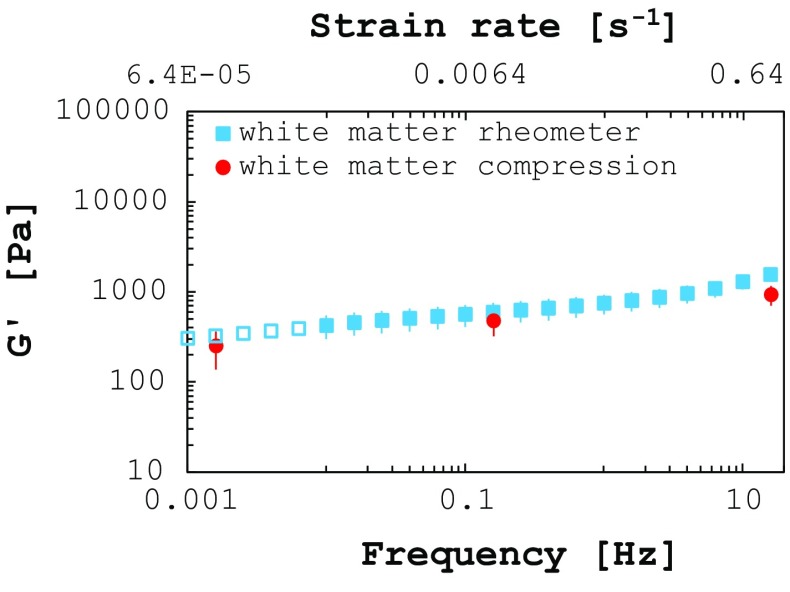
Comparison of white matter storage modulus measured by means of two different testing protocols

The results collected on white matter using the two test protocols were compared, and the results are shown in Fig. 6. The initial shear moduli (at small strains) were calculated from the average compression curves (Fig. 5c) and superimposed to the sweep frequency test results. The lowest frequency available for the rheometric measurements is 0.01 Hz (corresponding to $6.4 \times 10^{-4}\,\hbox {s}^{-1}$ strain rate). For this reason, a power law was used to extend the rheometric data points down to 0.001 Hz (hollow markers in Fig. 6; power exponent: 0.13; *R* value: 0.98). The results show good agreement and support the validity and consistency of the proposed testing protocol and adopted methodologies.

## Material parameters for brain constitutive model

The brain tissue was modelled as a hyper-viscoelastic “sponge-like” porous matrix saturated with CSF. Evidences have shown that the brain tissue follows the biphasic consolidation theory when subject to deformation and that a viscous component is also present in the solid matrix of the tissue, which behaves nonlinearly (Bilston et al. 1997; Cheng and Bilston 2007; Franceschini et al. 2006). The formulation allows the material behaviour to be divided into rate-independent and rate-dependent responses. These arise from the deformation of the hyperelastic solid network (rate-independent response), the viscous contribution of the solid network (rate-dependent response) and the flow of the liquid through this porous medium (second rate-dependent response). Similar or simplified formulations have been obtained in previous studies for porcine brain tissue (Cheng and Bilston 2007; Dumpuri et al. 2007; Miller 1999), but there is a lack of suitable parameters ready to be implemented in numerical codes to simulate human brain tissue. It is therefore important to derive and make available such parameters for both grey and white human matter.

Assuming that the medium is fully saturated, the total stress at a point, $\sigma$, is given by:

1 $$\begin{aligned} {\varvec{\sigma }} ={\bar{{\varvec{\sigma }}}}^{*}-p{\varvec{I}} \end{aligned}$$

where ${\bar{\sigma }}^{*}$ is the effective stress in the porous material skeleton, *p* is the pressure stress in the wetting liquid, and ${\varvec{I}}$ is the identity matrix. Considering the nonlinear elastic characteristics of brain tissue, the rate-independent response of the solid skeleton was assumed to follow the Ogden hyperelastic model. This model has a strain energy potential *U* defined as:

2 $$\begin{aligned} U=\frac{2\mu _0 }{\alpha ^{2}}\left( {\bar{\lambda }}_1^\alpha +\bar{\lambda }_2^\alpha +\bar{\lambda }_3^\alpha -3 \right) +\frac{1}{D}\left( {J-1} \right) ^{2} \end{aligned}$$

where ${\bar{\lambda }}_i$ are the deviatoric principal stretches and are equal to ${\bar{\lambda }} _i =J^{-\frac{1}{3}}\lambda _i$; $\lambda _i$ are the principal stretches; $\mu _0, \alpha$, and *D* are material parameters; and *J* is the volume strain (equal to $\lambda _1 \lambda _2 \lambda _3 )$. The stresses are then given by partial differentiation of Eq. (2), i.e. ${\bar{\sigma }}^{*}=\frac{\mathrm{d}U}{\mathrm{d}\lambda }$.

**Fig. 7:**
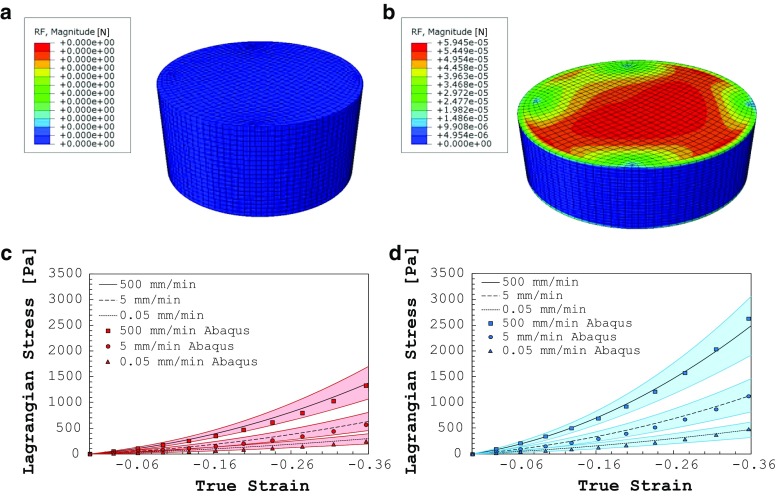
Numerical simulation of the uniaxial compression in undeformed (**a**) and deformed (**b**) configurations. The contour plot represents the nodal reaction forces. Compression results for grey (**c**) and white (**d**) human matter (24 $^{\circ }\hbox {C}$) and comparison with the Abaqus compression model results. The experimental measurements are depicted as *lines*, and the model results are depicted with *markers*

Note that $\mu _0$ is the instantaneous shear modulus, whereas the bulk modulus *K* is related to *D* and Poisson’s ratio, $\nu$, through:

3 $$\begin{aligned} D=\frac{2}{K}=\frac{3\left( {1-2\nu } \right) }{\mu _0 \left( {1+\nu } \right) } \end{aligned}$$

The rate-dependent response of the solid matrix is implemented in the model defining the shear stress $(\tau \left( t \right) )$ relation for a viscoelastic model:

$$\begin{aligned} \tau \left( t \right) =\mathop \int \nolimits _0^t \mu \left( {t-s} \right) {\dot{\gamma } }(s)\mathrm{d}s \end{aligned}$$

where $\dot{\gamma }$ is the shear strain rate and $\mu \left( t \right)$ is the time-dependent shear relaxation modulus which can also be written as:

4 $$\begin{aligned} \mu \left( t \right) =\mu _0 g_R \left( t \right) \end{aligned}$$

where $\mu _0$ is the instantaneous shear modulus mentioned above, which represents the shear relaxation modulus when $t=0$. Using a Prony series, one can obtain:

$$\begin{aligned} g_R \left( t \right) =1-\mathop \sum \limits _{i=1}^N g_i \left( {1-e^{-\frac{t}{\tau _i }}} \right) \end{aligned}$$

$g_i$ and $\tau _i$ are the Prony constants and the retardation time constants, respectively. Therefore, Eq. (4) becomes:

$$\begin{aligned} \mu \left( t \right) =\mu _0 \left( {1-\mathop \sum \limits _{i=1}^N g_i \left( {1-e^{-\frac{t}{\tau _i }}} \right) } \right) \end{aligned}$$

**Table 3:** Summary of the material coefficients in Abaqus format for implementing the poro-hyper-viscoelastic material formulation

Matter	Solid phase									Fluid phase		
	Rate-dependent						Rate-independent					
	$g_{1}$	$g_{2}$	$g_{3}$	$\tau _{1}$ (s)	$\tau _{2}$ (s)	$\tau _{3}$ (s)	$\mu _{0}$ (Pa)	$\alpha$	*D* (1/Pa)	*k* (m/s)	$e_{0}$	$\gamma _{\mathrm{w}}$ (N/m$^{3}$)
Grey	0.6	0.1	0.16	0.5	20	200	520	-4.4	1.3E−03	1.57E−9	0.2	9741
White							1030	-4.3	0.6E−03	1.57E−7		

The coefficients are given for both grey and white human matter

A minimum of three couples of constants ($g_{i}$ and $\tau _{i}$) was needed for replicating the rate-dependent response of the brain tissue. A first-order Ogden strain energy function was analytically fitted using the least mean square method with the experimental compression data. The initial shear modulus, $\mu _0$, and the parameter, $\alpha$, in Eq. (2) were obtained for the white and grey compression test curves at 24 $^{\circ }\hbox {C}$ (highest strain rate) and were assumed to provide a good estimate for the instantaneous shear modulus and parameters $\alpha$ for white and grey matter. The parameter *D* is required for the hyperelastic material formulation in order to correlate the Poisson’s ratio $\nu$ of the material to $\mu _0$ using Eq. (3). As suggested by Kaczmarek et al. (1997), $\nu = 0.35$ was the value selected for the Poisson’s ratio of the solid matrix and *D* was calculated accordingly. This value represents only the relative compressibility of the material’s solid phase, which allows fluid to be absorbed or exuded from the solid matrix (Taylor and Miller 2004). A liquid phase (incompressible by default, $\nu = 0.5$) is introduced in the formulation. The fluid flow is governed by Darcy’s law:

5 $$\begin{aligned} n{\varvec{v}}=-\frac{k}{\gamma _{\mathrm{w}} }\left( {\nabla p-\rho _{\mathrm{w}} {\varvec{g}}} \right) \end{aligned}$$

where ${\varvec{v}}$ is the fluid flow velocity vector, *n* is the porosity of the medium, $\gamma _{\mathrm{w}}$ is the specific weight of the fluid, $\nabla p$ is the pressure gradient vector, *k* is the hydraulic conductivity of the medium, $\rho _{\mathrm{w}}$ is the fluid density, and ${\varvec{g}}$ is defined as the gravitational acceleration vector:

$$\begin{aligned} {\varvec{g}}=-g\nabla z \end{aligned}$$

where *g* is the gravitational constant $({=}9.812\,\hbox {m}/\hbox {s}^{2})$ and *z* is the elevation above some data. Note that the hydraulic conductivity, *k* (units m/s), is related to conventional permeability, $\varPi$ (units $\hbox {m}/\hbox {s}^{2})$, through:

$$\begin{aligned} \varPi =k\frac{\eta }{\rho _{\mathrm{w}} g} \end{aligned}$$

As already mentioned, the brain tissue is assumed to be fully saturated with CSF, i.e. all voids in the material are filled up with the wetting liquid. In addition, the void ratio *e* is defined as the ratio of volume of wetting liquid $V_{\mathrm{w}}$ to the sum of the volumes of the solid $V_{\mathrm{s}}$ and trapped liquid $V_{\mathrm{t}}$:

$$\begin{aligned} e=\frac{V_{\mathrm{w}} }{V_{\mathrm{s}} +V_{\mathrm{t}} } \end{aligned}$$

Therefore, the porosity *n*, in Eq. (5), is related to void ratio, *e*, through:

$$\begin{aligned} n=\frac{e}{1+e} \end{aligned}$$

To complete the definition of the fluid phase, the liquid was treated as water, defining its specific weight $(\gamma _{\mathrm{w}})$. The permeability values (*k*) for grey and white matter were taken from Kaczmarek et al. (1997) and the initial void ratio of the tissue $(e_0 )$ from Nagashima et al. (1987). The definition of a biphasic model enables to capture the second rate dependency caused by the movement of the fluid in the solid matrix. The difference in the compressibility of the solid and liquid phases partially monitors this rate dependency as shown in Forte et al. (2015).

A finite element model of the uniaxial compression tests was designed using the poro-hyper-viscoelastic material formulation. The commercial software package Abaqus (Hibbett et al. 2013) was used for all analyses.

The grey and white matter compression specimens were simulated using a 3D cylindrical geometry representative of the tested samples (5.5 mm radius, 7 mm height) as shown in Fig. 7. Mesh convergence tests were performed to validate the final mesh density that was chosen when results deviated by less than 1%. A 8-node brick, reduced integration, hourglass control hybrid element, which includes pore pressure, was employed. The bottom surface was constrained in the direction of compression, while it was free to move and expand horizontally. A free draining condition was assigned to the outside surface to allow the fluid to exude out of the specimen. The top surface was displaced in the vertical direction such that the three different displacement rates used in the experiments were simulated, i.e. 500, 5 and 0.05 mm/min.

The Prony series constants ($g_{i}$ and $\tau _{i}$) were initially guessed by analytically fitting a third-order Prony series function with the rheological data (least mean square method) as suggested by Bergström (2005) and subsequently optimized to achieve the minimum discrepancy between the experimental stress–strain results, which have been used to characterize the rate-dependent behaviour of the organic tissue in compression, and the FEM simulation. Table 3 summarizes the final coefficients that were chosen, while Fig. 7 shows the comparison between the model results and the experimental compression data for grey (c) and white (d) matter. In order to obtain the Lagrangian stress shown in this figure, the nodal forces at the top surface were summed to obtain the total force *F* and divided by the initial top area of the cylinder.

## Conclusions

In the present work, two different experimental protocols were used for characterizing human grey and white matter and identifying the effects of testing conditions on the outcomes.

For DMA in particular, it has been noticed that if grip is assured between the rheometer plates and the specimen by means of sand paper, the effect of sample thickness is negligible. However, due the limited availability of the tissue, only three samples were devoted to this analysis, which is considered qualitative and preliminary at this stage. Nevertheless, the effect of sample thickness when testing the rheological properties of brain tissue had already been extensively reported by Nicolle et al. (2005). Although more samples are needed to draw solid conclusions on this aspect, our results are in agreement with the mentioned study.

Humidity and temperature play a major role in oscillatory analysis because of the duration of the test. In fact, human white matter can easily dehydrate and undergo sudden stiffening if humidity is not kept at high levels. The drying process is further enhanced at higher temperatures, leading to wrong measurements (storage modulus up to 21.9 times higher). Failing to control these testing conditions could have partially contributed to the spreading of results in the literature (Chatelin et al. 2010). Ageing does not seem to play a considerable role in the procedure.

**Fig. 8:**
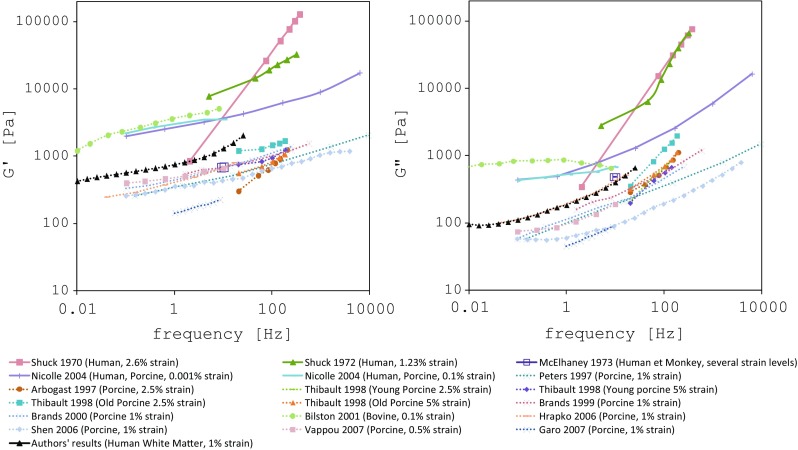
Authors’ results (in *black*) compared with previous findings. Human results are presented by continuous lines

Although care was taken to prevent dehydration throughout the test protocol, measuring the water content of the samples could have provided important additional information to the analysis. We therefore foresee the monitoring of the water content of the samples in future investigations and advise scientists to include this analysis when testing organic tissues. Furthermore, because of the high sensibility of the tissue to dehydration, errors in the results have also been noticed when using petroleum jelly to reduce moisture loss. Also, it should be noted that the storage and loss moduli become similar when testing samples using petroleum jelly. This might be due to the viscous effect introduced by the jelly on the measurements. In fact, in order to cover completely the sample and prevent dehydration, the jelly has to be distributed on the exposed edge of the organic specimen, forming a cylindrical shape around the tissue. The jelly therefore may contribute to the overall mechanical response of the sample. This leads to an increase of the measured loss modulus which approaches the storage modulus values over the frequency range hereby analysed. Additionally, the measured value for $G^{\prime }$ at the beginning of the test is identical for the moist controlled and the petroleum jelly tests (Fig. 4a). This proves that rehydrating the sample after the first run had the benefit of restoring the original tissue mechanical properties as also shown by a study on kidney tissue (Nicolle and Palierne 2010). This effect is also visible in Fig. 2b.

If the measurements are taken following a rigorous testing protocol, a softening of the tissue is clearly visible at higher temperatures, with $G^{\prime }$ and $G^{{\prime }{\prime }}$, respectively, 1.4 and 1.6 times lower if measured at body temperature (37 $^{\circ }\hbox {C}$) rather than at room temperature (24 $^{\circ }\hbox {C}$).

The population sample was not sufficiently wide to draw definitive conclusions on mechanical properties variation with gender. On the other hand, the authors can confidently state that for human brain tissue, different post-mortem times (between 26 and 48 h) did not affect the outcomes of the tests for the examined population, as previously proven for porcine brain tissue in some of the studies available in the literature (Darvish and Crandall 2001; McElhaney et al. 1973).

The noticeable difference in stiffness of grey and white matter confirms the results pointed out by MRE analysis (Bayly et al. 2012; Chatelin et al. 2010; Clayton et al. 2012) that showed white matter shear modulus to be 1.2–2.6 times higher than the one obtained for grey matter. In particular, our results show human white matter to be about 1.8 times stiffer than grey matter. The anisotropic behaviour of white matter was not investigated in the present study due to the presence of several fibre tracts oriented along different directions in the parietal lobe samples.

Figure 8 shows that the authors’ measurements (black lines) are within the spread of results generated by previous studies. Although our results seem to confirm that human tissue is stiffer than porcine tissue, they reveal a more complaint behaviour compared to the few human data available in the literature. However, some of these tests could have been run outside linear viscoelastic region (Shuck et al. 1970; Shuck and Advani 1972). In other cases, the mismatch remains uncertain (Nicolle et al. 2004, 2005). Nevertheless, the authors believe that the employment of two different mechanical testing protocols on human white matter tissue and the good correlation across the results strongly support the goodness of the data.

A complete poro-hyper-viscoelastic formulation was obtained for both grey and white matter. The material coefficients, whose use has been tested by simulating their suitability for reproducing the response of the tissue in a range of strain rates suitable to replicate surgical procedures, are readily implementable in numerical codes for finite element analysis of, for example, brain surgery procedures. Bilston and co-workers (Bilston et al. 1997; Cheng and Bilston 2007) showed that a poro-viscoelastic formulation provided a good match for the mechanical response of calve brain tissue. Similar results were obtained for liver tissue (Raghunathan et al. 2010). Additionally, Franceschini et al. (2006) demonstrated the importance of the fluid phase in the mechanical response of brain tissue and that viscous components are present in its solid phase. Our results show that a biphasic, nonlinear viscoelastic model (poro-hyper-viscoelastic formulation) is able to reproduce the rate-dependant response of human white and grey matter, in agreement with the previous findings.

The results hereby presented contribute towards understanding the complex and heterogenic mechanical properties of the human brain. The impact of the work is witnessed by the interest this topic generates across several fields of science, such as robotics, material science, tissue engineering and computer science at multiple levels. Knowing, simulating and mimicking the mechanics of the human brain could lead to the development of important research in several communities.
